# The association between dietary antioxidant quality score and intensity and frequency of migraine headaches among women: a cross-sectional study

**DOI:** 10.1186/s12905-024-03260-3

**Published:** 2024-09-09

**Authors:** Sara Hajishizari, Atieh Mirzababaei, Faezeh Abaj, Niki Bahrampour, Sajjad Moradi, Cain C.T. Clark, Khadijeh Mirzaei

**Affiliations:** 1 Department of Community Nutrition, School of Nutritional Sciences and Dietetics, Tehran University of Medical Sciences (TUMS), P.O.Box:14155-6117, Tehran, Iran; 2 Department of Nutrition, Science and Research Branch, Islamic Azad University (SRBIAU), Tehran, Iran; 3 Centre for Intelligent Healthcare, Coventry University, Coventry, CV1 5FB UK; 4 Victorian Heart Institute, Monash university, Melbourne, Australia; 5 Department of Nutrition and Food Sciences, Research Center for Evidence-Based Health Management, Maragheh, University of Medical Sciences, Maragheh, Iran

**Keywords:** DAQS, Migraine headache, Migraine disability, Visual analog scale

## Abstract

**Background:**

Migraine is an episodic disorder and a frequent form of headache. An impaired balance between free radical production and an impaired antioxidant defense system leading to oxidative damage may play a major role in migraine etiology. We sought to investigate whether dietary antioxidant quality score (DAQS) is associated with migraine intensity and frequency among women suffering from migraine.

**Methods:**

This cross-sectional study was conducted on 265 women. The data related to anthropometric measures and dietary intake were collected. DAQS score was calculated based on FFQ (food frequency questionnaire) vs. the reference daily intake (RDI) quantity. To measure migraine intensity, the migraine disability assessment questionnaire (MIDAS) and visual analog scale (VAS) were used. The frequency of headaches was defined as the days the participants had headaches in the last month and a 30-day headache diary was used.

**Results:**

The results of the study demonstrated that VAS, MIDAS, and frequency of headaches were reduced significantly from the low DAQS (poor quality of antioxidants) to high DAQS (high quality of antioxidants) after adjusting covariates. Also, multinomial regression showed there was an inverse association between higher DAQS and the frequency of headaches. In the adjusted model, subjects with the higher DAQS were 69% less likely to have moderate migraine disability, compared with those with the lower DAQS. Linear regression showed, there was an inverse association between vitamin C intake and the grades of pain severity.َAlso in a crude model, a negative association was found between vitamin E and the frequency of headaches.

**Conclusion:**

In conclusion, Participants with higher DAQS had lower migraine intensity and headache frequency. In addition, the consumption of vitamin C may potentially associate with decreasing the severity of headaches. Dietary antioxidants should be monitored closely in individuals suffering from migraine.

## Introduction

Migraine is a complex neurovascular inflammatory brain disorder that affects over 1 billion individuals across the world [1]. Migraine has been recognized as the greatest cause of disability in persons under the age of 50, affecting between 12 and 16% of the population, with women having a higher incidence than males (3:1), and it has a proclivity towards family occurrence [2–6]. It is a frequent form of headache and debilitating disease that is divided into two categories based on frequency. Chronic migraine (15 and more days per month) for at least 3 months and episodic migraine (less than 15 days per month) [7, 8]. Although considerable research has been done to understand the etiology of migraine headaches, the exact underlying mechanism is still unknown. Various causes have been postulated thus far, including neurogenic inflammation and trigeminovascular circuit activation [9]. However, there is mounting evidence that suggests the hypothalamus may be the trigger of migraine attacks [10]. As migraine attacks frequently follow a daily, monthly, or even seasonal rhythm, it is possible that the hypothalamic regions, which regulate the biological clock, have a role in the disease’s onset [11, 12]. Moreover, Brain imaging studies showed that during the very early phases of spontaneous migraine attacks, there is increased blood flow in the hypothalamic area [13].

Migraineurs are at a higher risk of cardiovascular disease and death [14]. As a result, figuring out the best way to treat and manage this condition is critical. Nonsteroidal anti-inflammatory drugs (NSAIDs) are by far the most used class of drugs for the acute treatment of headaches in general, and migraine in particular [15–17]. However, considering the potential for serious side effects from these drugs [18], identifying disease-modifying risk factors to avoid headaches is critical. Nutrition may have a role, according to research.

It was proposed that an impaired balance between free radical production and an impaired antioxidant defense system leading to oxidative damage may play a major role in pathological conditions including cancer, diabetes, hepatic disorders, cardiovascular disease (CVD), and neurodegenerative illnesses [19]. For decades, the concept of oxidative stress in migraine sufferers has been debated. The so-called nutraceuticals have received a lot of interest in recent years as compounds that may potentially be utilized to alleviate migraines [20]. Curcumin and coenzyme Q10, two antioxidants, were reported to reduce migraine frequency in previous studies [21, 22]. Vitamin E was found to reduce menstrual migraines [23]. Finally, previous investigations found that an antioxidant mixture of pine bark extract, vitamin C, and vitamin E reduced migraine symptoms [24, 25]. Antioxidants in food decrease oxidative stress by reducing the oxidative chain reaction’s start, dissemination, and completion. Scavenging free radicals, molecular oxygen quenching, and acting as reductants in oxidative processes are some of the various methods of action of antioxidants from food [26]. Furthermore, as previously stated, oxidative stress is thought to have a role in migraine etiology. Antioxidant supplementation can help to reduce the effects of oxidative stress [27].

Individual nutrients were the most often employed strategy for analyzing the possible role of antioxidant dietary consumption in health outcomes. This method, which focuses on the effects of a few specific antioxidants on health outcomes, leaves out a lot of data regarding the complicated or cumulative linkages and interactions that exist among antioxidant elements in foods [28]. The content and quantity of specific antioxidant components in the diet have been the most frequently used approach in establishing the possible influence of antioxidant dietary intake on health outcomes. The dietary antioxidant quality score (DAQS), which adds up the amounts of various dietary antioxidants and provides a score based on the computed quantity vs. the reference daily intake (RDI) quantity, has been proposed as a sensitive and accurate technique [28]. There is no available evidence regarding the association between DAQS and migraine severity, as far as we know. Thus, the purpose of this study was to assess the association of DAQS with migraine severity among Iranian females.

## Methods

### Study population

**Fig. 1:**
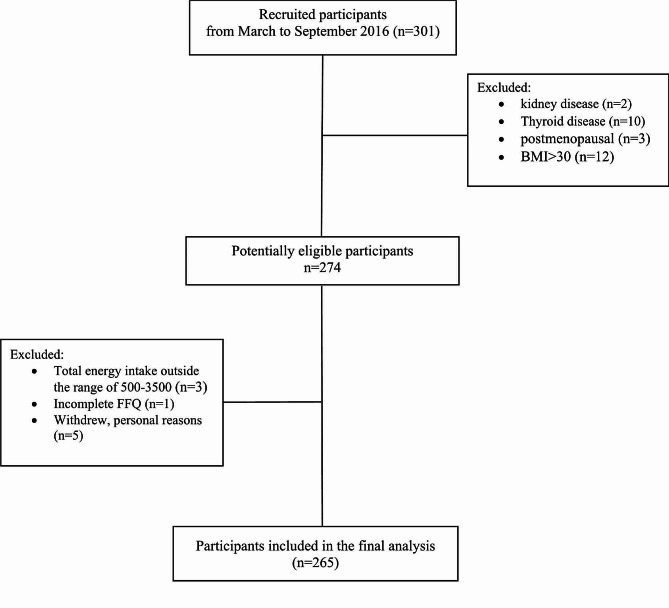
A flowchart showing participants through the study

We designed a cross-sectional study and finally enrolled 265 women who lived in Tehran, Iran, and had attended neurology clinics at two hospitals (Sina and Khatam Alanbia) and a professional headache clinic for migraine diagnosis from March to September 2016 (Fig. 1). The participants were selected based on the following inclusion criteria: women with the age range of 18–50 years, and BMI in the range of 18. 5–30 kg/m^2^, first visit in the headache clinic (had never been diagnosed with migraine, previously), and confirmation of migraine by a neurologist using the International Classification of Headache Disorders 3 criteria (ICHD3) [8]. We considered exclusion criteria included: having cardiovascular disease, liver, kidney, thyroid, cancer, diabetes, heart failure, and acute or chronic infections based on patient statements and medical history, consumption of drugs and supplements, pregnant, lactating, and postmenopausal women, drug and alcohol use, reluctance to continue reading were excluded. To control over- or under-reporting of food intake, subjects with daily energy intakes lower than 500 kcal or higher than 3500 kcal were excluded from the analysis. All procedures were followed in accordance with the ethical standards of the Tehran University of Medical Sciences (ethic number: 95-01-103-31348), which approved all aspects of the study. All participants signed a written informed consent prior to the start of the study.

### Migraine diagnosis

According to the headache classification committee of the International Headache Society (IHS) [29], an expert neurologist diagnosed migraine in the subjects. Two forms of migraine, with and without aura, are included in the criteria for diagnosing episodic migraine. According to the IHS, the following criteria can be used to make a diagnosis of migraine without aura: a headache with five or more bouts lasting 4–72 h; the headache should contain two or more of the following characteristics: unilateral, pulsating, moderate or severe pain intensity, worsened by or causing avoidance of regular activities, as well as one or more of the following signs and symptoms: nausea, vomiting, photophobia, and phono-phobia. Dizziness and vertigo, slurred speech, ataxia, tinnitus, visual disruption, and physical imbalance are all warning indications of migraine with aura [30].

### MIDAS and VAS questionnaires

The Migraine Disability Assessment questionnaire was used to measure migraine severity (MIDAS) [31]. This questionnaire has previously been translated and validated by Iranian people [32]. With five questions during the previous three months, this questionnaire assesses the severity of migraine headaches and their influence on patient performance. The patients were divided into four groups based on their total score on these five questions: Midas Grade I, Little or no disability (0–5); MIDAS Grade II, Mild disability [6–10]; MIDAS Grade III, Moderate disability [11–20]; and MIDAS Grade IV, Severe disability (21+).

In addition, pain intensity was measured using the VAS questionnaire. A VAS is usually a standard 100-mm visual analog scale (VAS) labeled No pain’ on the left side and Pain as intense as you can imagine’ on the right side. The participant marks on the line the point that they feel represents their perception of their current state. The VAS score is calculated by measuring in centimeters from the left-hand end of the line to the point that the patient marks. The following cut-off points present the severity of pain: mild pain [1–3], moderate pain [4–7], and severe pain [8–10, 33]. All Participants were asked to precisely complete a 30-day headache diary to collect information on the time of migraine attack onset, headache frequency and severity scores (based on VAS, from 0 to 10) precisely after each migraine attack no matter what time of day. The directions for completing the 30-day headache diary were provided by a qualified neurologist, even though individuals could contact researchers to resolve any issues while filling out their 30-day headache diary. At recruitment, the subjects were told to complete their headache diaries during the month ahead.

### Anthropometric measurements

Body weight was determined using a standard body weight scale (Seca 707; Seca GmbH & Co. KG., Hamburg, Germany). The participant’s height was measured, unshod, using a stadiometer (Seca GmbH & Co. KG.). To measure the waist-hip ratio, waist circumference (WC) in centimeters was divided by hip circumference in centimeters. We used a non-stretch tape measure to measure WC between the midpoint of the bottom ribs and the iliac crest hip bone following a normal exhale. Hip circumference was measured using a tape measure, while the participants were standing, at the point yielding the maximum circumference over the buttocks. Anthropometric measurements were applied with the minimum cloth and without shoes. The BMI was determined by dividing weight in kilos by height in meters squared.

### Dietary assessment

A person’s usual dietary intake over the past year was assessed by face-to-face interview using a semi-quantitative 147-item food frequency questionnaire (FFQ). It was administered by trained dieticians. Based on this questionnaire, the subjects were asked to report the frequency of their food consumption for each food item on a daily, weekly, monthly, or yearly basis. The reliability and validity of this questionnaire in Iran had already been confirmed [34]. Standard unit sizes and items reported on the household measures were converted to grams using the household measures Guide [35]. The energy content of the food items in the feed frequency questionnaire was determined using data from the USDA Food Ingredients Table in the Nutritionist 4 nutrition software database modified for Iranian foods (version 7.0; N-Squared Computing, Salem, OR, USA).

### Measurement of DAQS

DAQS was obtained from some vitamins and minerals that have antioxidant functions including selenium, zinc, vitamin A, vitamin C, and vitamin E [36]. To create a DAQS, we compared the daily intake of nutrients to that of the RDI [32]. Each of the 5 antioxidant intakes was assessed and then we allocated a value of 0 or 1, separately, for all components. According to Tur et al. [36] method when the intake was lower than 2/3 of the RDI, it was assigned a value of 0. Similarly, when the intake was higher than 2/3 of the RDI, it was assigned a value of 1. Thus, the total DAQS ranged from 0 (very poor quality) to 5 (high quality) [36] The percentage of the RDI as well as the proportion of individuals with intakes below the RDI, 2/3 of the RDI, and 1/3 of the RDI were calculated. The proportion of individuals with intakes below 2/3 of the RDI was the criterion used to estimate the risk of inadequate intake [37].

### Demographic characteristics

A demographic questionnaire was collected by researchers, containing questions about age, marital status, education, occupation, history of chronic disease, family history of migraine, drug consumption, and special diets. To assess the physical activity of the participants, the short form of the International Physical Activity Questionnaire (IPAQ) designed by the World Health Organization was used [38]. The validity and reliability of this tool have already been evaluated and accepted in Iranian adult women. The physical activity score is represented as metabolic equivalent (MET)/h/week.

### Statistical analysis

Data are presented as mean ± SD or frequency (%) for quantitative and qualitative, respectively. To evaluate the relationship between DAQS and the severity of migraine headaches, participants were categorized into 2 groups according to DAQS. To compare quantitative and qualitative variables across DAQS, the independent sample t-tests, and chi-square were used. The association between DAQS and migraine severity (MIDAS and VAS), and headache frequency (categorized into two groups: ≤15 days/month and > 15 days/month) were determined using multiple linear regression and multinomial regression. In the crude model MIDAS, VAS, and headache frequency, were entered into the model as response variables, and DAQS were entered as independent variables. In the adjusted model, the effects of age, weight, hip circumference (HC), job, education, physical activity, and energy intake were controlled. Data were analyzed using SPSS software version 24 (IBM Corp. IBM SPSS Statistics for Windows, Armonk, NY). *P* values ≤ 0.05 were considered statistically significant.

## Result

### Study population characteristics

265 subjects participated in the present study with mean age, height, weight, and BMI of 34.32 ± 7.86 years, 161.87 ± 5.15 cm, 69.41 ± 13.0 kg, and 26.50 ± 4.90 kg/m^2^, respectively as shown in Table 1. The MIDAS percentages of without, mild, moderate, and severe disability (based on the questionnaire) were 13.2, 24.9, 17.4, and 44.5%, respectively. Also, based on the VAS questionnaire, 16.2%, 42.9% and 41% of the study population had mild, moderate, and severe headaches, respectively. Besides, the frequency of the headache was less than 15 days/month in 62% and more than 15 days/month in 38% of the population.

**Table 1:** Characteristics of the total study population. Values are based on mean ± standard deviation

Variables	Mean ± SD (*n* = 265)	Median (*n* = 265)
Age(year)	34.32 ± 7.86	34.00
Height (cm)	161.87 ± 5.15	162.00
Weight (kg)	69.41 ± 13.0	68.00
BMI (kg/m^2^)	26.50 ± 4.90	26.57
WC (cm)	86.43 ± 12.72	85.00
HC (cm)	103.93 ± 12.20	104.00
WHR	0.87 ± 0.59	0.84
Physical activity (Met/h/week)	406.24 ± 519.73	280.00
VAS	6.34 ± 2.30	7.00
MIDAS	23.65 ± 19.87	19
Headache frequency	13.33 ± 9.48	12.00

WC: waist circumference; HC: Hip Circumference; WHR: waist to hip ratio; BMI: body mass index; MIDAS; Migraine Disability Assessment Questionnaire: VAS; Visual analog scale

### Association between population characteristics and DAQS

All participants were dichotomized based on DAQS. We assessed the differences in demographic variables between the low and -high-intake DAQS groups. Based on the results, higher DAQS was associated with lower HC (*p* = 0.03). The results of the comparison indicated that mean VAS and MIDAS were reduced significantly from the low DAQS to high DAQS, after adjusting for confounders (*p* < 0.05). Also, the frequency of headaches was reduced significantly from the low DAQS to high DAQS, moreover, the results remained significant after adjusting for confounders (*p* < 0.05). No differences were found in mean age, height, weight, BMI, WC, WHR, physical activity, education, job, and marital status (*p* > 0.05) between the low and high intake DAQS groups, even after adjusting for confounders, as shown in Table 2.

**Table 2:** Characteristics of the study population among DAQS

		DAQS		*P*-value	*P*-value*
		Low adherence (*n* = 148)	High adherence (*n* = 117)		
**Quantitative variables**					
Age(year)		34.04 ± 7.58	34.71 ± 8.23	0.49	0.32
Height (m)		162.07 ± 5.02	161.62 ± 5.33	0.47	0.40
Weight (kg)		69.75 ± 13.43	68.98 ± 12.73	0.63	0.99
BMI (kg/m^2^)		26.55 ± 4.96	26.43 ± 4.87	0.84	0.66
WC (cm)		85.34 ± 14.88	87.07 ± 12.23	0.31	0.97
HC (cm)		101.70 ± 14.87	105.44 ± 13.62	**0.03**	0.98
WHR		0.87 ± 0.54	0.88 ± 0.65	0.97	0.48
Physical activity (Met/h/week)		413.77 ± 411.58	386.63 ± 624.44	0.67	0.83
VAS		9.45 ± 15.08	7.72 ± 8.51	0.47	**0.01**
MIDAS		24.48 ± 19.09	22.73 ± 20.89	0.27	**0.02**
Headache frequency		14.47 ± 9.49	11.94 ± 9.32	**0.03**	**0.03**
**Qualitative variables**					
Education	Undergraduate	50	44.8	0.49	0.80
	Bachelor	31.8	27.6		
	Master or higher	18.2	27.6		
Job	Housekeeper	52.7	49.6	0.55	0.15
	Employed	47.3	50.4		
Marital status	Single	29.1	29.1	0.73	0.12
	Married	70.9	70.9		

Values are based on mean ± standard deviation or reported percentage. Significant items with a *P* value ≤ 0.05 are bolded. *P-value reported after adjusting for age, marital status, education, job, physical activity and energy intake

Independent sample t test for quantitative data and χ2 test for qualitative data have been used. Subjects in the low adherence of DAQS had DAQS (< 5); high adherence: (≥ 5). DAQS, dietary antioxidant quality score; WC, waist circumference; HC, Hip Circumference; WHR, waist to hip ratio; BMI, body mass index; MIDAS; Migraine Disability Assessment Questionnaire, VAS; Visual analog scale

### Association between dietary intakes and DAQS

The dietary intakes of the participants based on DAQS are shown in Table 3. The results of the comparison showed that the mean energy, protein, carbohydrate, total fat, cholesterol, vitamin B1, B2, B3, B6, D, E, A, folate, zinc, selenium, and magnesium were significantly higher in subjects with higher adherence to DAQS (*p* < 0.001). After adjusting for confounders including age, physical activity, and energy intake the mean carbohydrate, total fat, vitamin B2, D, E, A, and zinc remained significant (*p* ≤ 0.05).

**Table 3:** Dietary intake of nutrients among DAQS

Variables	DAQS		*P*-value	*P*-value^*^
	Low adherence (*n* = 148)	High adherence (*n* = 117)		
**Macronutrients**				
Energy (kcal/d)	1935.22 ± 406.16	2599.32 ± 394.34	**< 0.001**	**-**
Carbohydrates (g/d)	262.73 ± 55.91	329.01 ± 63.74	**< 0.001**	**< 0.001**
Protein (g/d)	73.17 ± 13.14	86.75 ± 15.80	**< 0.001**	0.62
Total fat (g/d)	71.79 ± 20.49	111.22 ± 27.88	**< 0.001**	**< 0.001**
Cholesterol (mg/d)	214.31 ± 74.69	286.84 ± 100.02	**< 0.001**	0.45
**Micronutrients**				
Vitamin B1 (mg/d)	1.51 ± 0.46	1.92 ± 0.48	**< 0.001**	**0.06**
Vitamin B2 (mg/d)	1.81 ± 0.44	2.05 ± 0.50	**< 0.001**	**0.04**
Vitamin B3 (mg/d)	20.53 ± 4.96	25.26 ± 5.54	**< 0.001**	0.66
Vitamin B6 (mg/d)	1.83 ± 0.39	1.96 ± 0.52	**0.02**	0.29
Vitamin C (mg/d)	136.42 ± 65.40	141.37 ± 109.44	0.64	**< 0.001**
Vitamin D (μg/d)	1.62 ± 0.92	2.38 ± 1.01	**< 0.001**	**0.03**
Vitamin E (mg/d)	8.92 ± 3.12	15.12 ± 4.33	**< 0.001**	**< 0.001**
Vitamin A (μg/d)	683.70 ± 287.16	753.43 ± 328.69	**< 0.001**	**< 0.001**
**Minerals**				
Folate (IU/day)	499.58 ± 123.59	563.10 ± 127.20	**< 0.001**	0.65
Se (μg/d)	92.78 ± 28.94	128.32 ± 39.60	**< 0.001**	0.51
Fe (mg/d)	43.54 ± 30.59	39.13 ± 27.41	0.22	**0.02**
Ca (mg/d)	1277 ± 486.61	1254.92 ± 461.89	0.70	0.09
Mg (mg/d)	370.27 ± 84.73	435.33 ± 138.76	**< 0.001**	0.07
Zn (mg/d)	10.37 ± 2.10	12.47 ± 2.90	**< 0.001**	**0.05**

Significant items with a *P* value ≤ 0.05 are bolded. *P -value reported after adjusting for age, physical activity and energy intake. The *p* value obtained from the independent sample t test. Subjects in the low adherence of DAQS had DAQS (< 5); high adherence: (≥ 5). DAQS, dietary antioxidant quality score; IU, international unit

### Association between MIDAS, VAS, and headache frequency with DAQS

The association between DAQS and migraine severity is shown in Tables 4 and 5. According to the analysis, the grades of pain severity were lower in subjects with higher adherence to DAQS. In the high-intake DAQS group, there was a lower percentage of participants with grade Ӏ and IV migraine disability though, there was no statistically significant difference across the two groups (*p* > 0.05). However, the Frequency of headaches was significantly lower in participants with higher adherence to DAQS (*p* < 0.05). Also, there was an inverse association between higher DAQS and the frequency of headaches (OR = 0.53, 95%CI = 0.31–0.88, *p* = 0.01). After controlling for confounding variables including age, physical activity, weight, energy intake, and job status of participants, the results remained significant (OR = 0.51, 95%CI = 0.25,1.04, *p* = 0.05). Individuals with higher DAQS were 49% less likely to have more than 15 days per month headaches (> 15 /month) compared with those with lower DAQS. Also, there was a relationship between moderate migraine disability and DAQS. In the adjusted model, subjects with higher DAQS were 69% less likely to have moderate migraine disability, compared with those with lower DAQS (OR = 0.31, 95%CI = 0.09–1.07, *p* = 0.05). Though, no relationship was observed between DAQS groups and mild and severe disability even after adjusting for potential confounders. In other words, there was no statistically significant correlation between higher DAQS and reduced disability in patients with mild disability (OR = 0.41, 95%CI = 0.13–1.27, *p* = 0.12), or severe disability (OR = 0.85, 95%CI = 0.29–2.49, *p* = 0.76). The analysis did not find any association between moderate pain, severe pain, and DAQS (OR = 0.52, 95%CI = 0.25–1.10, *p* = 0.08 and OR = 0.59, 95%CI = 0.28–1.24, *p* = 0.16, respectively). After adjusting for the effect of age, physical activity, weight, energy intake, and job status of participants as confounding variables the results remain insignificant.

**Table 4:** VAS, MIDAS and headache frequency among DAQS

		DAQS		*P*-value
		Low adherence (*n* = 148)	high adherence (*n* = 117)	
VAS^a^	Mild pain	29 (67.4)	14 (32.6)	0.22
	Moderate pain	59 (52.2)	54 (47.8)	
	Severe pain	60 (55.0)	49 (45.0)	
MIDAS^b^	Grade Ӏ	23 (65.7)	12 (34.3)	0.23
	Grade ӀӀ	32 (49.2)	33 (50.8)	
	Grade ӀӀӀ	22 (47.8)	24 (52.2)	
	Grade IV	70 (59.3)	48 (40.7)	
Headache frequency^d^	≤ 15 days/month	82 [49]	82 [49]	**0.01**
	> 15 days/month	66 (65.3)	35 (34.7)	

Values are based on n (%). χ2 test for qualitative data have been used. ^a^The VAS score was for mild pain [1–3], moderate pain [4–7], and severe pain [8–10]. ^b^MIDAS Grade I, Little or no disability (0–5); MIDAS Grade II, Mild disability [6–10]; MIDAS Grade III, Moderate disability [11–20]; and MIDAS Grade IV, Severe disability (21+). ^c^Headache duration, categorized as ≤ 10 h, less than or equal 10 h; >10 more than 10 h. ^d^Headache frequency, categorized as ≤ 15 days/month and > 15 d. Subjects in the low adherence of DAQS had DAQS (< 5); high adherence: (≥ 5). DAQS, dietary antioxidant quality score; VAS visual analog scale, MIDAS migraine disability assessment

**Table 5:** Relationship between VAS, MIDAS, and headache frequency with DAQS

	Crude Model			Adjusted Model^f^		
	OR	95% CI	*P*-value	OR	95% CI	*P*-value
VAS^a^						
Mild pain^b^	-	-	-	-	-	-
Moderate pain	0.52	0.25,1.10	0.08	0.65	0.25,1.72	0.39
Severe pain	0.59	0.28,1.24	0.16	0.85	0.31,2.32	0.76
MIDAS^c^						
Grade Ӏ^d^	-	-	-	-	-	-
Grade ӀӀ	0.50	0.21,1.18	0.11	0.41	0.13,1.27	0.12
Grade ӀӀӀ	0.47	0.19,1.18	0.11	0.31	0.09,1.07	**0.05**
Grade IV	0.76	0.34,1.67	0.49	0.85	0.29,2.49	0.76
Headache frequency^e^						
≤ 15 days/month	-	-	-	-	-	-
> 15 days/month	0.53	0.31,0.88	**0.01**	0.51	0.25,1.04	**0.05**

Significant items with a *P* value ≤ 0.05 are bolded. ^a^ The VAS score was for mild pain [1–3], moderate pain [4–7], and severe pain [8–10]. ^c^Midas Grade I, Little or no disability (0–5); MIDAS Grade II, Mild disability [6–10]; MIDAS Grade III, Moderate disability [11–20]; and MIDAS Grade IV, Severe disability (21+). ^e^Headache frequency, categorized as ≤ 15 days/month and > 15 d. ^b, d^ as a reference group. ^f^ adjusted for age, marital status, education, job, physical activity and energy intake; OR: odds Ratio; CI, confidence interval; DAQS, dietary antioxidant quality score, MIDAS; Migraine Disability Assessment Questionnaire, VAS; Visual analog scale

### Association between components of DAQS and MIDAS, VAS, and headache frequency

The association between migraine severity and antioxidant nutrients was examined using multiple regression analysis models adjusted by age, marital status, education, job, physical activity, and energy intake, and is presented in Table 6. The analysis showed that there was an inverse association between vitamin C intake and the migraine pain intensity (β= -0.18, 95%CI= -29.00, -5.72, *P* = 0.004). After adjusting for confounding variables, the results remained significant (*p* = 0.006).َAlso in a crude model, a negative association was found between vitamin E and the frequency of headache (β= -0.11, 95%CI= -4.41,0.15, *p* = 0.05). Moreover, DAQS had a significant negative association with headache frequency (β= -0.13, 95%CI= -4.82, -0.23, *p* = 0.03). After adjustment for confounding factors, the association remained significant (*p* = 0.05).

**Table 6:** The association of components of DAQS with MIDAS, VAS and headache frequency

	MIDAS			VAS			Headache frequency		
	**β (95% CI)	*P*-value	^*^P-value	**β (95% CI)	*P*-value	*P-value	**β (95% CI)	*P*-value	*P-value
Vitamin C (mg)	0.06 (-0.68,28.68)	0.29	0.14	-0.18 (-29.00, -5.72)	**0.004**	**0.006**	0.02 (-6.68,10.20)	0.68	0.69
Vitamin E (mg)	0.04 (-10.34,13.77)	0.77	0.72	-0.17 (-11.97,3.05)	0.24	0.11	-0.11 (-4.41,0.15)	**0.05**	0.15
Vitamin A (μg)	0.01 (-8.18,9.59)	0.87	0.79	-0.04 (-6.97,4.09)	0.87	0.82	0.06 (-1.50,4.59)	0.32	0.29
Zinc (mg)	-0.01(-5.01,4.59)	0.93	0.54	-0.01 (-0.59,0.52)	0.89	0.90	-0.06 (-3.58,0.98)	0.26	0.33
Selenium (μg)	0.02 (-3.67,5.93)	0.64	0.22	0.03 (-0.38,0.73)	0.54	0.81	-0.07 (-3.69,0.88)	0.22	0.35
DAQS	-0.09 (-16.98,9.36)	0.57	0.31	0.12 (-4.99,11.41)	0.44	0.81	-0.13 (-4.82, -0.23)	**0.03**	**0.05**

Significant items with a *P* value ≤ 0.05 are bolded. **β coefficient obtained from linear regression

^*^Adjusted for age, marital status, education, job, physical activity and energy intake

DAQS, dietary antioxidant quality score, MIDAS; Migraine Disability Assessment Questionnaire, VAS; Visual analog scale. CI, confidence interval

## Discussion

This is the first research to investigate the relationship between DAQS and migraine headaches among women based on our knowledge and literature search. A significant inverse association was found between DAQS and headache frequency after adjusting for confounders. Individuals with higher DAQS scores were 49% less likely to have more than 15 days per month headaches (> 15 days/month) compared with those with lower DAQS. Furthermore, the mean score of VAS, MIDAS, and headache frequency were reduced significantly from the low DAQS to the high adherence of DAQS.

One of the processes involved in migraine etiopathogenesis is thought to be oxidative stress, which is regarded as changes in the balance between ROS production and degradation. It has been known for years that oxidative stress plays a role in the pathogenesis of migraines [39, 40]. By providing antioxidants, the impact of oxidative stress may be modulated [27]. Additionally, the medications now being used to prevent migraines do have some antioxidative activity [27].

In the present study, we have demonstrated a negative association between vitamin C intake and migraine pain intensity. Furthermore, vitamin E was also inversely correlated with headache frequency. In line with this study, Ferroni et al. study emphasized the critical role of antioxidant agents as a dietary intervention due to reducing the brain oxidative redox system [27]. Additionally, Chayasirisobhon et al. in an uncontrolled open-label study found that receiving 60 mg of vitamin C, and 30 International Units (IU) of vitamin E can improve both headache frequency and headache severity in patients suffering from migraine [41].

**Fig. 2:**
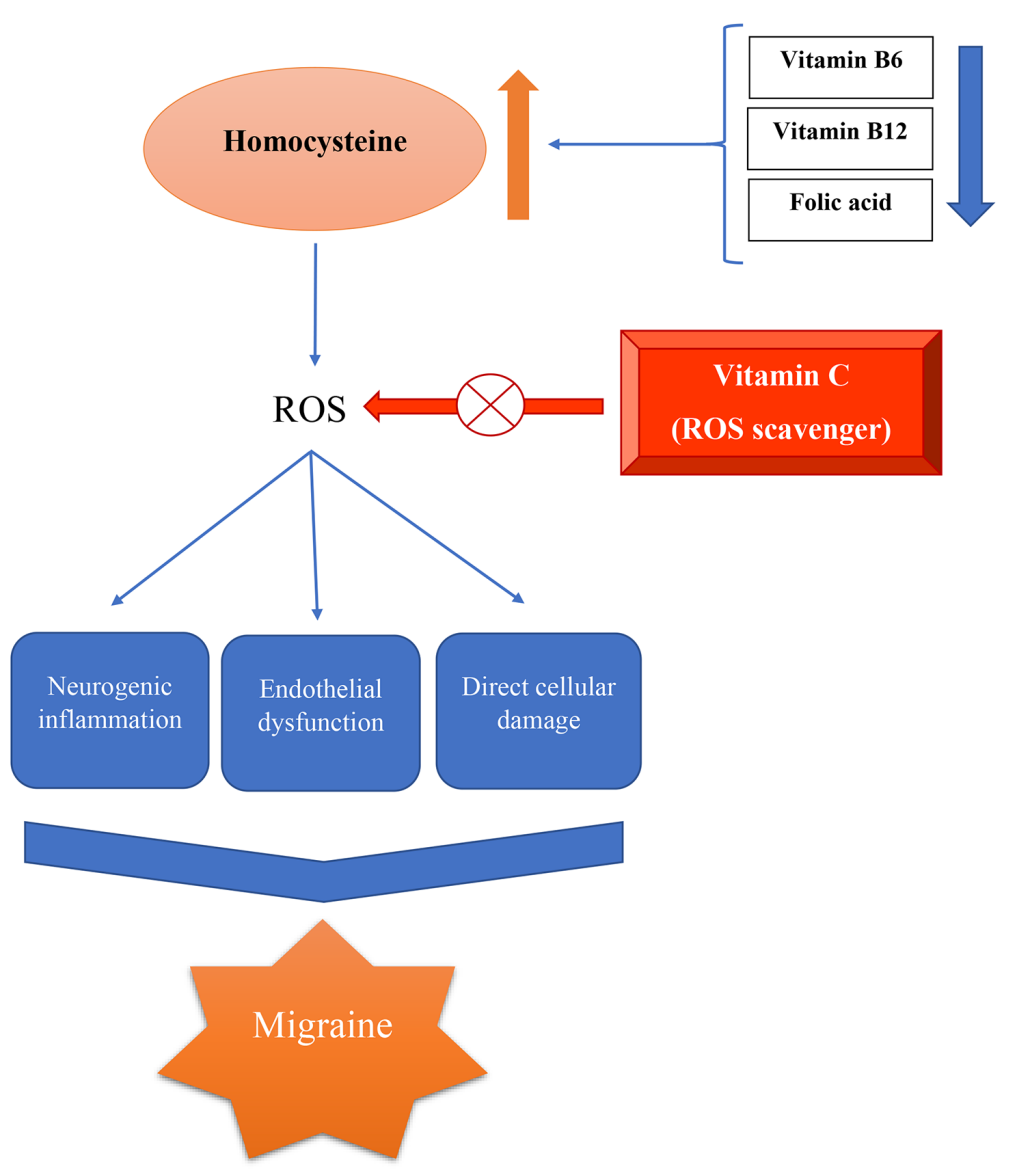
Schematic representation depicting the possible roles of vitamin C in migraine pathophysiology. ROS: Reactive oxygen species

To date, no randomized controlled trial (RCT) has been conducted to examine the effectiveness of vitamin C as a preventative therapy for migraine. However, the findings of different studies in which vitamin C was administered following wrist or ankle injury, as a daily dose from 200 to 1500 μg, led researchers to hypothesize that consumption of vitamin C, which is a ROS scavenger and an antioxidant, may also modulate the effects of neuroinflammation and ROS activity during migraine [42, 43] Fig. 2. Apart from supplements, plant-based foods such as fruits, vegetables, flowers from edible plants, and spices are excellent dietary sources of natural antioxidants [44, 45]. Polyphenols, Carotenoids, and vitamins C and E are the most prevalent plant antioxidants [46–48].

In our study, we found that vitamin E was also inversely associated with headache frequency. In accordance with our study, Ziaei et al. demonstrated that there was a decline in the pain severity and improvement in the functional disability scales among female migraineurs who utilized vitamin E for five days during their menstruation periods [49].

**Fig. 3:**
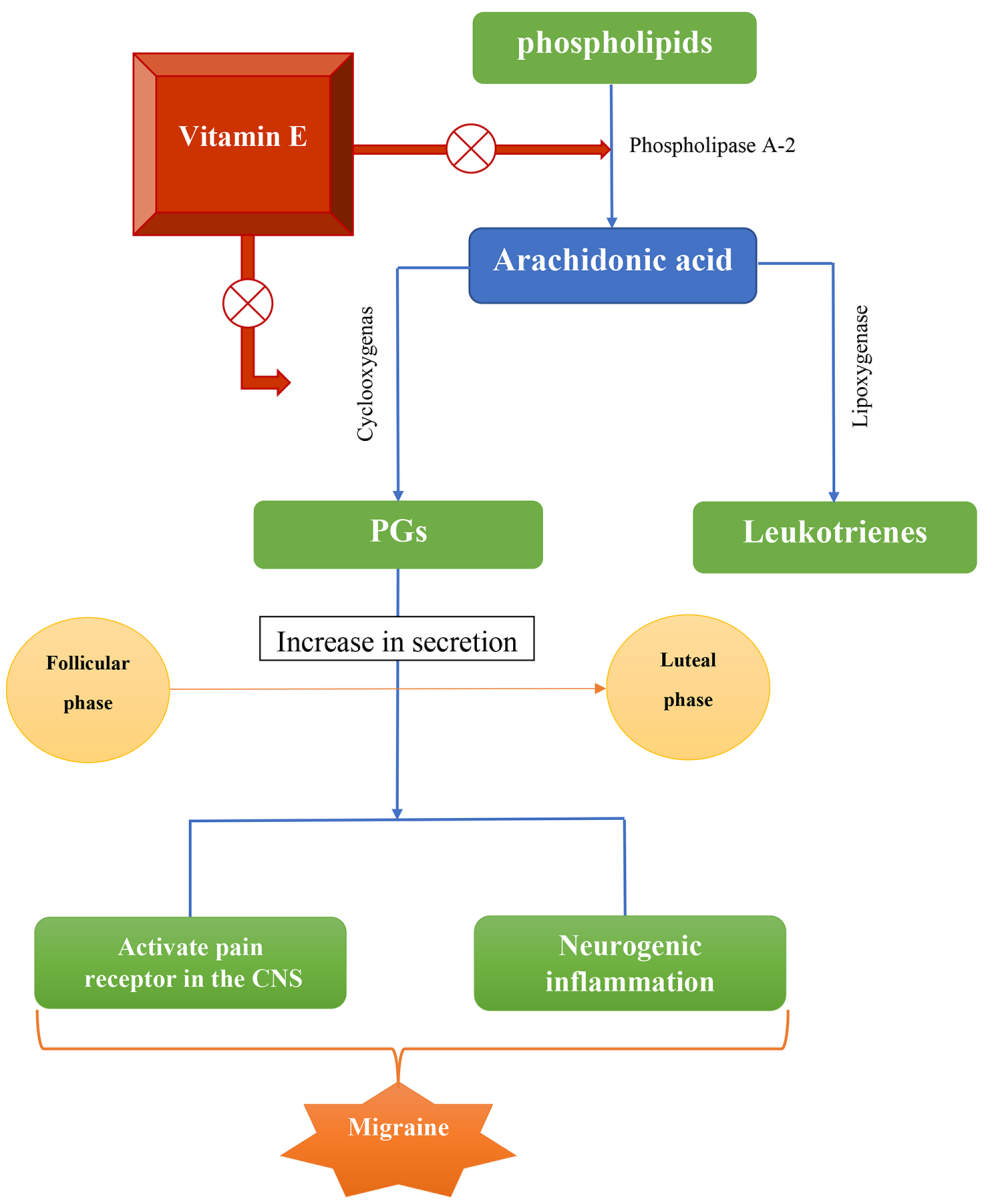
Schematic representation depicting the possible roles of vitamin E in relation to PGs as prophylaxis of menstrual migraine. CNS: central nervous system and PG: prostaglandins. Red circled times symbol: inhibition of the pathway

Vitamin E, an anti- prostaglandins substance with relatively few side effects, is useful for reducing migraine symptoms and headache pain [49, 50]. Additionally, it lessened the requirement for rescue drugs and functional disability [49, 50]. Vitamin E may be effective by inhibiting the release of arachidonic acid and producing prostaglandins. The enzymes phospholipase A2 and cyclooxygenase will be blocked by vitamin E, especially in menstrual migraine headaches [51] Fig. 3.

In addition, a previous study reported that following a nutrient pattern full of calcium, vitamin A, vitamin K, vitamin C, vitamin B6, vitamin B2, and magnesium may reduce the severity of headaches [52]. In addition, one review study which was aimed to investigate the role of nutrients in the pathogenesis and treatment of migraine headaches did not find any improving effects of nutrients except magnesium, carnitine, riboflavin, niacin, CoQ10, vitamin D, vitamin B12, and alpha lipoic acid [53]. In contrary with this study, one study found that selenium administration has a protective effect on mice brains by the antioxidant phenomena [54]. So, the results are in the ways of the previous study.

In the present study, we showed that DAQS had a significant negative association with headache frequency. Individuals with higher DAQS were 49% less likely to have more than 15 days per month headaches (> 15 days/month) compared with those with lower DAQS.

Antioxidants supplied with food prevent oxidative stress by inhibiting initiation, propagation, and the oxidative chain reaction itself. Other mechanisms that antioxidants from food act, include scavenging free radicals, quenching molecular oxygen, and functioning as reductants in oxidative processes [26].

It may be due to decreasing tissue damage and microvascular dysfunction after following high DAQS intakes [51]. Antioxidants may reduce reactive oxygen species (ROS) due to preventing produce of neuropeptides such as substance P (SP) and calcitonin gene-related peptide (CGRP) [51]. We should consider that the mean (SD) of intakes of vitamin A, C, zinc, and selenium were equal to or higher than the recommended daily allowances (RDA) in the both lower and higher median of DAQS [55]. A double-blind randomized placebo-controlled clinical trial found that zinc supplementation which has a role in neuronal signaling, can reduce the frequency of migraine attacks but not the duration and severity of cold-type migraine headaches [56]. In this study, some of the insignificant results may attribute to the type of headaches. Overall, a cross-sectional study found that reducing the total intake of food may have a better influence regardless of the type of food [57]. This can justify our results.

One of the limitations of this study is the failure to consider different types of headaches. Second, the cross-sectional design of the study can only examine the relationship, not casual effects. Third, we did not consider foods like caffeine that trigger migraine. Fourth, we did not assess the menstrual time of participants which may have effects on the severity of migraine. Finally, a larger sample size is needed to increase the accuracy of the results. DAQS and pain intensity data are based on questionnaires and interviews with patients, which are subjective and based on patients’ memory and their interpretation of pain. This was the first study to investigate the relationship between DAQS and headaches in migraineurs. The population was free of any chronic diseases, and this can reduce the effects of confounders.

## Conclusion

Our study found that higher consumption of DAQS nutrients may reduce headache frequency among women. In addition, the consumption of vitamin C may potentially associate with decreasing the severity of headaches. Also, a higher DAQS score was related to lower moderate migraine disability. Although this study did not present a significant relationship between all DAQS subcategories and migraine headaches severity, it should be considered that having a balanced diet full of vegetables and antioxidants alongside maintaining a normal weight is proven to reduce headaches. It is evident that more prospective studies are needed to confirm the veracity of our results.

## Data Availability

The authors confirm that the data supporting the findings of this study are available within the manuscript and in the included tables.

## Abbreviations

BMI: Body Mass Index
CI: Confidence Interval
DAQS: Dietary Antioxidant Quality Score
FFQ: Food Frequency Questionnaire
HC: Hip Circumference
IPAQ: International Physical Activity Questionnaires
IU: International Unit
MET: Metabolic Equivalents
MIDAS: Migraine Disability Assessment
NSAIDs: Nonsteroidal anti-inflammatory drugs
RDA: Recommended Daily Allowances
RDI: Reference Dietary Intake
VAS: Visual analog scale, WC, waist circumference
WHR: Waist to hip ratio
